# Compounded medication for patients with rare diseases

**DOI:** 10.1186/s13023-017-0741-y

**Published:** 2018-01-04

**Authors:** Marc Dooms, Maria Carvalho

**Affiliations:** 1 University Hospitals Leuven, Herestraat, B 3000 Leuven, Belgium; 2 PCCA, Houston, TX USA

**Keywords:** Pharmaceutical compounding, Extemporaneous preparations, Unlicensed medicines, Compounded medications, Orphan drugs, Rare diseases, Official preparations, Magistral preparations

## Abstract

**Background:**

When there is no authorized on- or in absence even no off-label treatment for patients with rare diseases, pharmacists have to compound medicinal products to meet the patients special needs. However it is important that there is evidence in the medical and/or pharmaceutical literature for such compounded medications.

**Position statement:**

Pharmaceutical compounding must be performed in the best possible circumstances by certified practitioners (pharmacists) using validated standard operating procedures (standardized formulations) in order to obtain medicinal products of the highest quality to assure patient safety. More than 60 compounding procedures were identified in 17 on-line pharmaceutical compounding reference sources worldwide but more operating procedures still need to be validated. All ingredients used in the preparation of the compounded medication must be accompanied by a certificate of analysis and full records of the pharmaceutical production process need to be kept for full traceability and accountability.

## Background

Pharmaceutical compounding corresponds to the preparation of unlicensed medicines in order to meet specific patient needs that do not have a licensed (commercial) medicine available on the market. It is no alternative to a licenced orphan drug that follows the usual pathway of development and marketing authorization but compounding is rather complementary if there is no interest from sponsors in the marketing of such a product. More research and development, targeting new pathways and exploring new pharmacological treatment is needed for the designation and authorization of orphan drugs. Patients with rare diseases have individual needs that may benefit from sterile and non-sterile compounded medication since there is a limited number of 144 orphan drugs currently authorised by the European Medicines Agency (EMA). Furthermore, “one-size” does not fit all patients and the existing orphan drugs may not always be adequate regarding the pharmaceutical dosage form or dosage strengths, raw materials (hypersensitivity reactions) or the organoleptic characteristics. For instance, it is common practice to prepare a sildenafil oral liquid instead of dispensing the licensed tablets (Revatio®) for paediatric patients with pulmonary arterial hypertension and the licensed oestradiol (Progynova®) dosage strength often needs adjustments in females with Turner syndrome. Pharmaceutical compounding may also be the only treatment option when the orphan drugs are discontinued or become temporarily unavailable, such as the shortage of ibuprofen injection (Pedea®), the mecasermin solution for subcutaneous injection (Increlex®) in 2013 and arsenic trioxide injection (Trisenox®) in 2017.

In the past, all medicines were compounded formulations prepared *secundum artem*^1^ and it was not until the early 1900s, with the advent of industrialisation and drug manufacturing, that the commercial medicines gradually dominated the pharmaceutical market. At that time, the practice of compounding did not cease to exist but declined substantially worldwide. Nowadays, as we move towards a pharmacogenomics-driven approach to treatment, compounded medication are resurging as an invaluable alternative for optimized treatment outcomes [1] and decreased drug-related adverse effects (intolerance and hypersensitivity reactions). In the United States and Brazil, there are currently compounding-only pharmacies that prepare thousands of compounded medication per day. In the European Union (EU), compounded medication are traditionally prepared extemporaneously in community and hospital pharmacies for one particular patient in accordance with a medical prescription. Notwithstanding this, compounded medication may also be prepared in advance (of a patient’s request), in batches (of variable sizes), and in other settings apart from a pharmacy (e.g. the specials manufacturers in the UK) [2].

There are several professional organizations around the world dedicated to the promotion, support and education of pharmaceutical compounding, as examples are displayed in Table 1. The majority of these organizations represent the national interests of compounding pharmacists and membership is usually required. Some of these organizations hold training courses and seminars throughout the year, which are essential for the continuous education of compounding pharmacists. Compounded medication for rare diseases are commonly discussed in these events as, for instance, at the recent ‘IX Symposium: Unification of criteria for paediatric formulations’ by Aprofarm (Table 1) in Seville, Spain [3]. Apps with compounding formulations are not available (except for “FagronFormulas”) yet but twitter accounts are all over.

**Table 1:** Examples of professional organisations dedicated to pharmaceutical compounding

Country		Organisation	Website	Membership
ARGENTINA	FORMULAR	*Asociación Civil de Farmacéuticos Formulistas Argentinos*	http://www.formular.org.ar/	Yes
BRAZIL	Anfarmag	*Associação Nacional de Farmacêuticos Magistrais*	http://www.anfarmag.com.br/	Yes
CANADA	ACPC	Association of Compounding Pharmacists of Canada	http://acpcrx.org/	Yes
FRANCE	SOTP	*Société des Officinaux sous-Traitants en Préparations*	http://www.preparationmagistrale.fr/	No
ITALY	SIFAP	*Società Italiana Farmacisti Preparatori*	https://www.sifap.org/	Yes
SPAIN	AFA	*Asociación de Formulistas de Andalucía*	http://www.formulistasdeandalucia.es/	Yes
SPAIN	AEFF	*Asociación Española de Farmacéuticos Formulistas*	http://www.aeff.es/	Yes
SPAIN	Aprofarm	*Asociación Profesional Independiente de Farmacéuticos Formuladores*	http://www.aprofarm.org/	Yes
USA	IACP	International Academy of Compounding Pharmacists	http://www.iacprx.org/	Yes
WORLDWIDE	ISPhC	International Society of Pharmaceutical Compounding	http://www.isphc.org/	Yes

## Compounding legislation

The EU pharmaceutical legislation, Directive 2004/27/EC (amending Directive 2001/83/EC on the Community code relating to medicinal products for human use), allows member states to supply compounded medication, namely magistral and officinal formulas, in accordance with the legislation in force and to fulfil special needs. As referred to in Articles 3 [4, 5], a magistral formula is “any medicinal product prepared in a pharmacy in accordance with a medical prescription for an individual patient”; an officinal formula is “any medicinal product which is prepared in a pharmacy in accordance with the prescriptions of a pharmacopoeia and is intended to be supplied directly to patients served by the pharmacy in question”. The EU member states transposed the EU directive and adapted it to their national jurisdictions. As a result, the definition and regulation of pharmaceutical compounding is not harmonized in the EU [6]. For instance, in addition to the EU definition of magistral and officinal formulas, there is the concept of pharmacies’ own formulas in Finland and the specials in the UK [2]. With regards to regulations, third-party compounding (outsourcing of compounded medication) is an example of a practice that is not permitted in Poland, whereas it is a frequent practice in France and Portugal. In some EU Member States pharmacists can eventually outsource all the compounding to other pharmacies or so-called special-order manufacturers (https://www.bnf.org/ page 944–945), especially when the procedure is considered to be too difficult (advanced therapy medicinal products) or dangerous (cytotoxic and radio-active agents and vaccinations).

## Compounding quality and risks

The quality, safety and efficacy of compounded medication is not guaranteed by randomised clinical trials, as it is for the licensed (commercial) medicines, though health must not be compromised when patients are dispensed both stock and extemporaneous preparations. The EU Resolution CM/Res(2016)1 on quality and safety assurance requirements for medicinal products prepared in pharmacies for the special needs of patients [succeeding Resolution CM/Res(2011)1] highlights the need for quality preparations Europe-wide and sets a list of principles to be adopted by the national frameworks, inter alia an appropriate quality assurance system and a model procedure for risk assessment. As stated, for the “high-risk preparations” it is recommended that the Good Manufacturing Practices (GMP) guidelines [7] are used as reference, whereas for the “low-risk preparations” the PIC/S PE 010–4 guide [8] may be used instead, or other guidelines with an equivalent level of quality [6].

Despite the recommended quality guidelines, compounded medication have endangered public health in unfortunate events worldwide, some with devastating repercussions. Intoxication after percutaneous resorption in babies and small children (skin thickness different from adults) with rare skin diseases has been reported for erroneously prescribed salicylic acid [9, 10], urea [11, 12] and lactic acid [13]. Side-effects have also been published due to an accidental overdose caused by a compounding pharmacy error [14] or by switching from one chemical form to the other [15].

Pharmaceutical compounding carries significant risks but, if the principles listed above are considered and medication are prepared in accordance to standardized formulations, the benefits of administering compounded medication outweigh the potential risks [16]. The EU Resolution CM/Res(2016)1 preconizes that stock preparations should be required product dossiers that compile all necessary information to justify the standardized formulation and method of preparation. For extemporaneous preparations, such product dossiers are not required since “it could lead to a delay in the supply of necessary medicines” [6]. However, extemporaneous preparations should also be prepared in accordance to standardized formulations, even if considered ‘low-risk’. Though pharmacists may not prepare a product dossier in a timely manner for extemporaneous preparations, there are many compounding reference sources online that list standardized formulations of interest, as displayed in Table 2. These reference sources include official national formularies and pharmacopoeias, scientific journals and structured databases, which have been compiled by professional organizations, pharmaceutical companies, hospital pharmacies and independent pharmacists. Some of these reference sources are available online at no cost whereas others have a subscription fee – an investment that compounding pharmacists should considered in order to access an official national formulary or a large international database such as the *CompoundingToday* [17]. In addition to these reference sources that list standardized formulations in a structured manner, there are also online forums and blogs that address useful compounding information, such as the Spanish *Consulta de Formulación Magistral de Acofarma* [18]. As a result, compounding pharmacists have nowadays access to many online compounding sources that may be used as reference for the extemporaneous preparations which, although do not have a product dossier compiled at the pharmacy, these may still be prepared with assured quality and safety.

**Table 2:** Online pharmaceutical compounding reference sources

Country	Type	Online reference source	Website	Availability
Belgium	National formulary	*Therapeutisch Magistraal Formularium*	https://www.tmf-ftm.be	Free
Canada	Online database	Children’s Hospital of Eastern Ontario	http://www.cheo.on.ca/en/healthcareprofessionalsforpharmacists	Free
Canada	Online database	Compounding Recipes Index	http://www.sickkids.ca/Pharmacy/Compounding-Service/	Free
Canada	Online database	IWK Compounding Formulas	http://www.iwk.nshealth.ca/page/iwk-compounding-formulas	Free
France	National formulary	*Formulaire National*	http://ansm.sante.fr/Mediatheque/Publications/Pharmacopee-francaise-Formulaire-national	Free
New Zealand	Online database	eMixt	http://www.pharminfotech.co.nz/manual/Formulation/mixtures/	Free
Spain	Online database	*Formulación en Farmacia Pediátrica*	http://www.manuelaatienza.es/03_formulacion.htm	Free
Spain	Online database	*Formulas Magistrales de la SEFH*	http://gruposdetrabajo.sefh.es/farmacotecnia/index.php?option=com_content&view=article&id=32&Itemid=19	Free
Spain	Online database	*WikiSpace de Formulación Magistral*	https://formulacion-magistral.wikispaces.com/INICIO	Free
Switzerland	Online database	*CHUV Produits Fabriqués*	http://www.chuv.ch/pharmacie/pha_home/pha-prestations/pha-production-pharmaceutique/pha-produits-fabriques-2.htm	Free
Switzerland	Online database	*Préparations Magistrales Dermatologiques en Suisse*	http://www.magistralrezepturen.ch/	Free
Switzerland	Online database	OpenAPO	https://www.openapo.info/	Free
United States	Journal	U.S. Pharmacist: Pharmacy Practice, Compounding	https://www.uspharmacist.com/topic/compounding	Free
United States	Online database	Compounding Matters	https://www.fagron.com/en/knowledge/compounding-matters	Free
United States	Online database	Michigan Pediatric Safety Collaboration	http://www.mipedscompounds.org/standard-formulations	Free
United States	Online database	Nationwide Children’s	http://www.nationwidechildrens.org/outpatient-pharmacy-compounding-formulas	Free
Country	Type	Online reference source	Website	Availability
Germany	National formulary	*Deutscher Arzneimittel-Codex Neues Rezeptur-Formularium*	http://dacnrf.pharmazeutische-zeitung.de/	Subscribers only
Netherlands	National formulary	*Formularium Nederlandse Apothekers*	https://www.knmp.nl/producten-en-diensten/fna-boek-2013	Subscribers only
Slovenia	National formulary	*Formularium Slovenicum*	http://www.formularium.si/	Subscribers only
United States	National formulary	United States Pharmacopeia (USP) Compounding Compendium	http://www.usp.org/store/products/usp-compounding-compendium	Subscribers only
United Kingdom	National pharmacopoeia	British Pharmacopoeia	https://www.pharmacopoeia.com/	Subscribers only
United States	National pharmacopoeia	United States Pharmacopeia (USP) – National Formulary (NF)	http://www.usp.org/store/products/usp-nf	Subscribers only
United States	Journal	International Journal of Pharmaceutical Compounding	https://www.ijpc.com/	Subscribers only
United States	Online database	CompoundingToday	http://compoundingtoday.com/	Subscribers only

The quality and safety of compounded medication also relies on the active pharmaceutical ingredients and excipients used in the formulations as for authorized orphan drugs. When these are registered pharmaceutical compounds [19] with a monograph in the European Pharmacopoeia (for example creatin, dapsone, glycine, hydroxocobalamine, ketoconazole, l-carnitine, oxybutynin, primaquine phosphate) or any other pharmacopeia (for examples 3,4-diaminopyridine, pyridoxal phosphate) a pharmaceutical analysis of the ingredients can be performed and a certificate of analysis delivered. When there is no monograph yet (for example bi-myconase, cholic acid, l-citrulline, diphencyprone, mepacrine, uridine), the chemical, pharmaceutical and microbiological quality of the ingredients should be demonstrated on the basis of validated methods [6, 20]. A certificate of analysis is an essential document in any compounding procedure.

## Compounded medication for rare diseases

The recent EU Commission Report (2016/C 424/03 of 18 November 2016) [21] describes more in detail the criteria for designation of an EMA orphan drug namely the expression “a similar medicinal product as a satisfactory method authorized in the Union” as follows:

*“In certain cases, medicinal products prepared for an individual patient in a pharmacy according to a medical prescription, as referred to in Article 3(1) of Directive 2001/83/EC (commonly known as the ‘magistral formula’), or according to the prescriptions of a pharmacopoeia and intended to be supplied directly to patients served by the pharmacy, as referred to in Article 3(2) of that Directive (commonly known as the ‘officinal formula’), may be considered as satisfactory treatment if they are well known and safe and this is a general practice in the EU. If the product proposed for designation is not authorized to be placed on the market, patients in the EU may still be treated with it if it is prepared in a pharmacy”.*

The establishment of “significant benefit in relation to the medicines that are part of the standard of care of the target rare disease” is essential in the authorisation of a new orphan medicine in the EU.

Compounded medication is now considered as an existing “satisfactory treatment” for whom significant benefit needs to be proven.

Pharmacists already compounded medicinal products for their patients with rare disorders long before they became authorized orphan drugs in the EU [22] such as 3,4-diaminopyridine capsules (Firdapse) [23], 5-aminolevulinic acid drink (Gliolan), betaine drink (Cystadane), caffeine citrate injection (Peyona), cholic acid capsules (Kolbam, Orphacol), cysteamine bitatrate capsules (Cystagon, Procysbi) and eyedrops (Cystadrops) [24], ibuprofen injection (Pedea), p-aminosalicylic acid capsules (Granupas), and zinc acetate capsules (Wilzin). These extemporaneous preparations were made in accordance with an individual medical prescription (“Fac secundum artem”) eventually with a formulation published in a pharmacopeia or according to other references. Several excellent review articles have been published with compounding formulation for the treatment of patients with genodermatosis such as ichthyosis [25].

Sterile [26] and non-sterile compounding for patients with rare diseases is routine pharmaceutical practice today: infusion bags for total parenteral nutrition (neonates with inborn errors of metabolism); medicines and placebos for phase 1 clinical trials with designated orphan drugs; radioactive and other drugs with stability limitations in PK/PD studies for rare disorders; (repurposed) medicaments awaiting orphan drug authorization or withdrawals (drug shortages); masking of taste and smell in medicines for babies with metabolic disorders; and medicinal products for special populations such as paediatrics [27].

Examples of standardized formulations (Standard Operating Procedures) to be used in patients with rare diseases and already published are the listed in Table 3.

**Table 3:** Standardized compounded medication commonly prescribed for rare diseases

Compounded Medication	Information Source
Beta-Carotene 30 mg & 100 mg capsules	TMF
Cafeine without citrate 1% 10 ml injection	OpenAPO
Cafëine-citraat 20 mg/2 ml injection	BP, FNA, IJPC
Chenodeoxycholic acid 250 mg capsules	TMF
Ciclosporin 2% eye drops	IJPC
Cysteamine 0,15% & 0,5% eye drops	DAC, FFP
Glycopyrrolate 0,5 mg/ml oral suspension	CT, Fagron, IJPC, IWK, NZ
Hydroxocobalamine 0,1% 10 ml injection	BP, OpenAPO
Levo-Arginine HCl 100 mg/ml 50 ml injection	FNA
Levo-Arginine HCl 100 mg/ml oral liquid	TMF
Levo-Carnitine 200 mg/ml oral liquid	CT, TMF
Midazolam 0,5% nasal spray	FFP
Midazolam 2,5% nasal spray	OpenAPO
Penicillamine 50 mg/ml oral suspension	Fagron
Polihexanide 0,02% eye drops	OpenAPO
Potassium−/sodiumcitrate oral liquid	FNA (prep), IJPC
Potio Joulie (disodium hydrogenphosphate) oral liquid	HUG, IJPC
Primaquine phosphate 30 mg capsules	TMF
Primaquine phosphate 6 mg/5 ml oral suspension	MPA
Propranolol 1 mg/ml oral suspension	Fagron, FFP, IJPC, IWK, SEFH
Propranolol 5 mg/ml oral suspension	CHEO, FPP, NZ
Pyridoxal phosphate 10 mg capsules	TMF
Pyridoxal phosphate 25 mg/ml oral suspension	NC
Riboflavin 10 mg/ml oral suspension	CRI, Fagron
Sildenafil Citrate 2,5 mg/ml oral suspension	CHEO, IWK, MPA, NZ, SEFH, USP40
Sodium benzoate 100 mg/ml oral liquid	TMF
Sodium dihydrogen phosphate 156 mg/ml oral liquid	CT, FNA
Sodium phenylbutyrate 200 mg/ml oral liquid	IJCP, TMF, USP40
Sodium thiosulphate injection	BP
Ursodeoxycholic acid suspension 15 mg/ml oral suspension	FFP
Ursodeoxycholic acid suspension 20 mg/ml oral suspension	IWK, MPA, NC
Ursodeoxycholic acid suspension 50 mg/ml oral suspension	CHEO, CRI, NZ, USP40

## Discussion

The safety and efficacy of most of the treatments with compounded medication are not based on randomised clinical trials establishing the safety and efficacy but on evidence collected during years of clinical practise. Compounded medicinal products are therefore dispensed without a package insert but some pharmacists and/or formularies developed their own package inserts as pharmaceutical care is very important for a better care to the patients. Compound specific databases comprise ChEMBL, ChemSpider, PubChem, OpenPHACTS and the Orange Book with supportive information on a compound’s chemistry and pharmacology.

Drug shortages for orphan (Pedea) and non-orphan drugs (Ammonaps) did appear [28] and compounding was necessary then to continue the life-saving treatment. For other drug shortages (Cerezyme, Fabrazyme) compounding was not possible because the active ingredient was not available and patients didn’t get their proper treatment as long as the shortage took. When a drug is taken from the market by the sponsor (sometimes used off-label), pharmacists had to compound the medicinal product to continue the treatment of their patients with rare disorders. For example Mexitil was taken from the market by the pharmaceutical company and substituted by compounded capsules with mexilitine to continue the successful treatment of patients with myotonic disorders. Fludrocortisone capsules to treat patients with Addison syndrome are not (anymore) on the market in every EU Member State and therefor need to be compounded in some countries. Patients awaiting the marketing and reimbursement of repurposed medicines [29–31] were treated during this process by compounded preparations (propranolol for infantile haemangiomas).

Stability of compounded medicinal products has been published elsewhere (http://www.stabilis.org/) but is always more problematic for medicinal products produced by the pharmaceutical industry as extemporaneous preparations are used almost immediately after production. When intended for injectable use, extensive stability information can be found in the Handbook of Injectable Drugs (http://www.ahfsdruginformation.com/handbook-on-injectable-drugs/).

The cost for the patient is mostly lower for compounded preparations than the rather high cost of an authorized (orphan) drug [32–34]. A European legislation is in place to regulate standardized pharmaceutical compounding [4, 5] but local regulations still need to be installed for other aspects such as the reimbursement of the compounded medicinal products and the authorization for the use of some active and non-active ingredients. Especially for those that are on the WHO Model List of Essential Medicines for Children such as dapsone, fludrocortisone, hydroxocobalamin and primaquine. A Royal Decree in Belgium will regulate this from November 1^st^ 2017.

A new area in pharmaceutical compounding has arrived with Advanced Therapy Medicinal Products (cell therapy, gene therapy and tissue engineering) with his specific legal framework and Commision-of-Advanced-Therapies classifications. Several EU Member States installed the hospital exception to allow hospital pharmacies to produce these innovative medicines for their hospitalized patients. This will require cooperation with specialized personnel often outside the pharmacy such as blood banks and cell labs. Standardization in human stem cell therapy is also needed. Furthermore a protein bedside-manufacturing model on demand and at a small scale has been described for proteins [35] providing just enough drug for one single patient. This could open new possibilities for ultra-orphan drugs and individualized medicines. Finally 3D printed medicinal products incorporating biomaterials, 3D printed medicines and 3D bioprinting, still experimental and in need of EU legislation (such as biological tissues, organs and cells for medical applications), are compounded today in third line hospitals [36]. Personnel need to be trained and equipment installed to perform these new compounding procedures to obtain medicinal products of the best possible quality.

## Conclusions

When there is no on-label or even no off-label treatment for patients with rare diseases pharmacists have to compound the medication. This needs to be done in the best possible conditions by trained compounders following validates procedures.

Desktop research revealed that several validated standard operating procedures for the compounding of medicinal products for patients with rare diseases are already published in different compounding formularies. Medical prescriptions for such a treatment should mention the reference of the formulary for more harmonization as the same medical prescription needs to result in the dispensing of the same compounded medicinal product in all hospital and community pharmacies. Shared pharmacy work documents need to be followed to compound a medicinal product conform to all the quality standards of the European Pharmacopoeia. All ingredients used in the compounding must be accompanied by a certificate of analysis and full records of the pharmaceutical production process need to be kept for full traceability and accountability.

In this way medicinal products of the best possible quality will be dispensed to our patients with rare diseases. “When it’s not in the book, it’s up to the cook”.

## Abbreviations

ChEMBL: Chemical Database of Bioactive Molecules, European Bioinformatics Institute
ChemSpider: Chemical Databes, Royal Society of Chemistry
CHEO: Children’s Hospital of Eastern Ontario
CRI: Compounding recipes index
CT: Compounding today
DAC: Deutscher Arzneimittel-codex (Formulary German Pharmacists)
EMA: European Medicines Agency
EU: European Union
FFP: Formulation en Farmacia Pediatrica
FNA: Formularium Nederlandse Apothekers (Formulary Dutch Pharmacists)
FPP: Formulation in pharmacy practice
GMP: Good manufacturing practice
HUG: Hôpitaux Universitaires de Genève
IJPC: International Journal Pharmaceutical Compounding
IWK: IWK Health Center Canada
MPA: Michigan Pediatric Safety Collaboration, Standardization of Oral Liquids
NC: Nationwide Cildren’s Hospital, Columbus, Ohio, USA
NZ: New Zealand eMixt
OpenAPO: Formulary Swiss Pharmacists
OpenPHACTS: Discovery Platform for Drug Discovery
Orange Book: Approved Drug Products, Food and Drug Administration
PIC/S: Pharmaceutical Inspection Co-operation Scheme
PubChem: Open Chemistry Database, National Center for Biotechnology Information
SEFH: Sociedad Espanola de Farmacia Hospitalaria
TMF: Therapeutisch Magistraal Formularium (Formulary Belgian Pharmacists)
UK: United Kingdom
USP40: United States Pharmacopoeia 40th edition

## Footnotes

1 *Secundum Artem* (according to art): in accordance with the rules of the art (*Oxford English Dictionary*. 2nd edn. [Online]. Oxford University Press).

## References

[1] Koch J, Mayr JA, Alhaddad B, Rauscher C, Bierau J, Kovacs-Nagy R, Coene KL, Bader I, Holzhacker M, Prokisch H, Venselaar H, Wevers RA, Distelmaier F, Polster T, Leiz S, Betzler C, Strom TM, Sperl W, Meitinger T, Wortmann SB, Haack TB (2017). CAD mutations and uridine-responsive epileptic encephalopathy. Brain.

[2] Carvalho, M. Extemporaneously Compounded Oral Medicines in European Hospital Pharmacies. Doctoral thesis, UCL (University College London); 2013.

[3] Aprofarm, IX Symposium: Unification of criteria for paediatric formulations – Programme leaflet. Available at: http://www.formulistasdeandalucia.es/ficheros/9472%20triptico%20Pediatria.pdf Accessed: 21 May 2017.

[4] European Parliament and the Council (2001). Directive 2001/83/EC of 6 November on the community code relating to medicinal products for human use. Off J Eur Communities.

[5] European Parliament and the Council (2004). Directive 2004/27/EC of 31 march amending directive 2001/83/EC on the community code relating to medicinal products for human use. Off J Eur Union.

[6] Council of Europe (2016) Resolution CM/ResAP(2016)1 on quality and safety assurance requirements for medicinal products prepared in pharmacies for the special needs of patients [Online]. Available: https://www.edqm.eu/sites/default/files/resolution_cm_res_2016_1_quality_and_safety_assurance_requirements_for_medicinal_products_prepared_in_pharmacies.pdf Accessed: 20 May 2017. 10.1136/ejhpharm-2016-001017 PMC6451559 31157793

[7] European Commission (2016) ‘The rules governing medicinal products in the European Union: EU guidelines for good manufacturing practice for medicinal products for human and veterinary use’, *Eudralex*, Volume 4. Available at: https://ec.europa.eu/health/documents/eudralex/vol-4_en. Accessed: 20 May, 2017.

[8] PIC (2014) PIC/S Guide to good practices for the preparation of medicinal products in healthcare establishments (PE 010–4) [Online]. Available at: https://www.picscheme.org/layout/document.php?id=156. Accessed: 20 May 2017.

[9] Martinez JLV, Stanescu S, Bustamante SC, Del Rey SJ, Macarron CP, Perez AC (2015). Unrecognized transcutaneous severe salicylate intoxication in an infant. Ped Emerg Care.

[10] Madan RK, Levitt J (2014). A review of toxicity from topical salicylic acid preparations. J Am Acad Dermatol.

[11] Beverley DW, Wheeler D (1986). High plasma urea concentrations in collodion babies. Arch Dis. Childhood.

[12] Garty BZ (1986). High plasma urea concentration in babies with lamellar ichthyosis. Arch Dis Child.

[13] Ramirez MJ, Youseef WF, Romero RG, Martinez JMQ, Gonzalez-Ensenat MA, Vilaplana XS, Cubells CL (2006). Acute percutaneous lactic acid poisoning in a child. Ped Dermatol.

[14] Schwam E (2011). Severe accidental overdose of 4-Aminopyridine due to a compounding pharmacy error. J Emerg Med.

[15] Becker ML, Al Hadithy AFY, Van den Bemt PMLA, Hunfeld NGM (2013). Switching to different genetic medicines: a checklist for safety issues. Eur J Hosp Pharm.

[16] Lutz E, Pauletti G, Carvalho M, Davidson G, Ashworth L, Subramaniam V, Llambí F (2014). The role of compounding in closing therapeutic gaps-abstracts from FIP 2013: when not to compound and considerations before compounding. Int J Pharm Compd.

[17] CompoundingToday: Compounding Formulas. Available at: http://compoundingtoday.com/Formulation/. Accessed: 4 June 2017.

[18] Acofarma: Consulta de Formulación Magistral de Acofarma. Available at: http://www.formulacionmagistral.org/. Accessed: 3 June 2017.

[19] Rowe RC, Sheskey PJ, Cook WG, Fenton ME (2012). Handbook of pharmaceutical excipients.

[20] Selmin F, Casiraghi A, Minghetti P (2016). Council of Europe Resolution and Pharmacy Compounding. Hospital Pharmacy Europe Suppl: Purchasing for Safety.

[21] Commission notice on the application of Articles 3,5 and 7 of Regulation (EC) No 141/2000 on Orphan Medicinal Products. Official Journal of the European Union 2016/C 424/03 of 18 November 2016.

[22] Minghetti P, Giudici E, Montanari L (2000). A proposal to improve the supply of orphan drugs. Pharmacol Res.

[23] Quartel A, Turbeville S, Lounsbury D (2010). Current therapy for Lambert Eaton myastenic syndrome: development of 3,4-diaminipyridine phosphate salt as first-line symptomatic treatment. Curr Med Res Op.

[24] Roy S, Descamps F, Planas V, Caudron E, Husson MC, Do B (2011). Cysteamine eyedrops: optimisation of the manufacturing process and meeting safety and efficacy criteria for continuous supply. Int J Clin Pharm.

[25] Traupe H, Burgdorf HC (2007). Treatment of Ichthyosis: there is always something you can do. J Am Acad Dermatol.

[26] Ndri H, Husson MC, Trouvin JH (2011). Injectable sodium benzoate: uses in France and future prospects. Int J Clin Pharm.

[27] Nunn AJ (2003). Making medicines that children can take. Arch Dis Child.

[28] Jaroslawski S, Azaiez C, Korchagina D, Toumi M (2016). Quantifying the persisting orphan-drug shortage public health crisis in the United States. J Mark Access Health Policy.

[29] Selmin F, Musazzi UM, Cilurzo F, Minghetti P (2017). Alternatives when an autorized medicinal product is not available. Medicine Access @ Point of Care.

[30] Padhy BM, Gupta YK (2011). Drug repositioning: re-investigating existing drugs for new therapeutic indications. J Postgrad Med.

[31] Davies EH, Fulton E, Brook D, Hughes DA (2017). Affordable orphan drugs: a role for non-for-profit organizations. Br J Clin Pharmacol.

[32] Dooms M, Pincé H, Simoens S (2013). Do we need authorized orphan drugs when compounded medications are available?. J Clin Pharm Ther.

[33] Simoens S, Cassima D, Picavet E, Dooms M (2011). Are some orphan drugs for rare diseases too expensive? A study of Purches versus compounding costs. Drugs Ther Perspect.

[34] Heemstra H, Cornips MCA, de Meijer M, Rietdijk CD, Slot TK, Meulenhoff PCW & Leufkens HGM. Hospital pharmacy compounding of orphan drugs. European Association of Hospital Pharmacists 13th conference paper Feb 2008.

[35] Schellekens H, Aldosari M, Talsma H, Mastrobattista E (2017). Making individualized drugs a reality. Nat Biotechnol.

[36] Liaw C-Y, Guvendiren M (2017). Current and emerging applications of 3D printing in medicine. Biofabrication.

